# 

**Published:** 1845-12

**Authors:** 


### WESTERN JOURNAL

OF

### MEDICINE AND SURGERY.

### EDITORS.

---

DANIEL DRAKE, M. D.,

PROFESSOR OF PATHOLOGY AND THE PRACTICE OF MEDICINE
IN THE MEDICAL INSTITUTE OF LOUISVILLE:

LUNSFORD P. YANDELL, M. D.,

PROFESSOR OF CHEMISTRY AND PHARMACY IN THE SAME:

THOMAS W. COLESCOTT, M. D.

### TO OUR CORRESPONDENTS.

In finishing our tenth volume, and at the close of the year, we may be permitted to devote a page to those who are, or should be, our correspondents. To the former we beg leave to return our thanks, and hope to hear from them hereafter. To the latter, a much larger number, what shall we say that we have not already said and reiterated? Why, we say, that they are unfaithful to themselves, to us, and to the profession, in not from time to time, committing to record on our pages, some of their daily observations. Not an hour elapses, from year to year, without bringing forth new and important facts, in some parts of our extended country; which from not being published are forever lost to science and humanity. In many instances this omission is the result of habits of indiscrimination or indolence, which every physician should be anxious to correct. In other cases the omission comes from excess of modesty, the individual feeling, or fearing, that nothing he could write would be worthy of publication. This might sometimes be well founded, if nothing but essays or dissertations could be published; but such papers, although most difficult of execution, are in general of least value—are least appropriate to a vehicle like ours, designed to announce original facts and observations, rather than discuss principles. We respectfully appeal to the class of physicians to whom we are now speaking, by the question, whether a week ever elapses, without their meeting with new facts—aspects of disease and effects of remedies—which, if made public, would advance our science and benefit society? We hope and trust, they will take this question to heart, and hereafter act under its suggestions. Now, that we are about to commence a new volume, *we* propose to act, as though *they* would act as we have recommended. We shall, in the editorial head set apart, under the title of—FACTS FROM CORRESPONDENTS,—or some other caption, a number of pages, in which to record the isolated observations of those whom we are addressing. Being thus presented, no one need to fear that they will not be read and appreciated by all our subscribers; and every one may rely, on their being extracted or noticed, by other journals when they are new and important.

We cannot dismiss this brief appeal, without stating a fact, which shows conclusively the backwardness of our brethren in the matter of writing. The three hundred and forty students of medicine, which make up the present class of the Institute, have not brought us a line for the Journal, although their departure was calculated to suggest to their preceptors and medical friends, the idea of contributions by them. Most ardently do we hope that this will not again be the case.

In conclusion, as this number will reach many of our readers, when the holidays are at hand, we beg leave to offer them the compliments of the season; and trust, that when they have collected their yearly accounts, and find themselves in good spirits and with but little to do, they will not forget that they have an annual contribution of their experience to make to the profession, and that we shall gladly aid them in the discharge of that duty. EDS.

---

### ALABAMA MEDICAL UNIVERSITY.

### THE NATURALIST.

Such is the title of a monthly publication projected by the Faculty of Franklin College, near Nashville, in Tennessee, which is to be devoted to

### INDEX TO VOL. IV.

[NEW SERIES.]

A

Abscess, mammary, treatment of, 173.
Abscess in the groin, 265.
Acephalous monster, 401.
Air, admission of, into veins, 538.
Alabama medical university, 546.
Aloes, extract of, 345.
American Journal of science and art, 272.
Anatomy and diseases of the breast, by Sir A Cooper, 412.
Anemia, 525.
Aneurism, 537.
Apoplexy, 527.
Arm, paralysis of, 444.
Arsenic, use of, 346.
Arsenic, blood an antidote for, 181.
Arteries, Neil on, 450.
Astonishing scientific discovery, 182.

B

Baker, Dr., a case of alleged monomania. 368.
Belladonna and naphtha in gout, 449.
Belladonna, cases of poisoning by 429.
Black-tongue, 277.
Blood, state of, 345.
Blood, antidote for arsenic, 171.
Bloodless amputation, 92.
Boston Medical and Surgical Journal, 274.
Bougies of slippery elm bark, 433.
Breast, anatomy of; by Sir A. Cooper, 412.
Breast pump in mammary abscess, 173
Buchanan's address, 369.
Buck's lectures on diseases of the chest, 16, 185,
Byrne's analysis, &c., 246.

C

Capshaw on epidemic erysipelas, 1.
Calomel, symptoms of poisoning by, 256.
Carbonate of lead in burns, 160.
Carbonic acid exhaled from the lungs, 449.
Causes of diarrhœa, 191.
Cherry kernels, poisoning by, 173.
Chest, lectures on diseases of, 16, 185.
Chemistry for students, Fowne's, 274,
Chemistry, Simon's, 497.
Children, gangrene of mouth in, 245.
Chlorosis, cause and treatment of, 174.
Chorea St. Viti, 535.
City, health of, 184.
Civilization, effect of, on disease, 441.
Clinical lectures, 353.
Climate in treatment of diabetes, 450.
College, Rush Medical, 367.
Colles's lectures on surgery, 211.
Colon, intus-susception of, 80.
Colon, inflammation of, 297
Commentaries of Heberden, 437.
Congenital malformation of the heart, 438.
Consumption cured, 328.
Consumption, spontaneous cure of, 270
Cooper, Sir A., on the breast, 412.
Cooper's, Samuel, surgery, 420.
Correspondents, 545.
Creation, vestiges of, 233, 308.

D

Dawson, on epidemic erysipelas, 93.
Dawson on diseases of Ohio, 461.
Disease, decrease of, from civilization, 441.
Dentition, 257.

Death of Dr. Houghton, 542.
Dental surgeons, association of, 454.
Dental surgery, principles, &c., of, 319.
Diabetes, climate in treatment of, 450.
Diarrhœa, 191, 511.
Dick's observations on dyspepsia, 164
Dickson's essays, 543.
Dictionary of medical terms, 428.
Diseases of the chest, 16, 185.
Diseases and hygiene of females, 37.
Double consciousness, 261.
Dudley's case of tumor on the thigh 254.
Dyspepsia, various forms of, 164

E

Encysted placenta, 408.
English epitome of Hippocrates and Galen, 462.
Emery's cases, 410.
Emetics in exhaustion, 263.
Epidemic erysipelas, 93, 285.
Epidemic in Henderson county, Ky. 198.
Epidemic metritis, 386.
Ergot of Rye, 538.
Erysipelatous fever, 1.
Erysipelatous laryngitis, 277.
Extract of green walnut in throat diseases, 328.
Extract of aloes, pills of 345.

F

Fee's case of encysted placenta, 408
Females, diseases of, 37.
Fistula in ano, 516.
Foreign bodies in the system, 533.
Fowne's chemistry, 274.
Fossil remains, 256.
French and English opinions of typhus, 264.
Fungous diseases of the gums, 405.

G

Galen, epitome of, 462.
Gangrene of the mouth in children, 245.
Gastrotomy, case of, 250.
Gastric fever, 328.
Geneva, medical matters in, 434.
Gordon's account of epidemic metritis, 386.
Gout, belladonna in, 449.
Groin, abscess in, 265.
Gross's pathological anatomy, 542.
Gross on the use of carbonate of lead, 160.
Gums, fungous disease of, 405.
Gunshot wound, case of, 304.
Gunshot wounds of the hip, 175.

H

Hahnemann and his system, 356.
Harris's Dentistry, 319.
Harris on diarrhœa, 191.
Harrison's therapeutics and materia medica, 459.
Harrison's materia medica, 483.
Harvard University, medical department of, 275.
Harvey's case of injury of the leg, 306.
Heart, malformation of, 438.
Heberden's commentaries, 475.
Hepatic abscess, death from, 267.
Higginbotham on ipecac in exhaustion, 263.
Hip, injury of, 14.
Hip, gun-shot wounds of, 175.
Hippocrates, epitome of, 450.
Hoblyn's dictionary, 428,
Homœopathy in Italy, 155.
Homœopathy, secrets of, 359.
Houghton, Dr., death of, 543.
Howel's case of abscess, 265.
Human fœtus, deformity of. 271.
Hydrocyanic acid, poisoning by, 524.

I

Improved life preserver, 458.
Inflammation of the colon, 217.
Inaugural address of Dr. Buchanan 369.
Infantile gastric fever, treatment of, 450,
Injury of the hip, case of, 11.
Insanity, occupation in, 262.
Insanity, surgical operation in a case of, 259.
Ipecauanha in exhaustion, 263.
Intermarriage of the races, Dickson on, 91.
Intus-susception of the colon, 80.
Irwin's case of inflammation of the colon, 297.
Italy, homœopathy in, 155.
Jaundice, 509.

K

Keller on erysipelatous laryngitis, 277.

L

Leg, injury of, 346.
Lectures on diseases of the chest, Buck's 16, 185.
Lectures on surgery, Colles', 211.
Lectures, clinical, 353.
Lecture on modern improvements in surgery, 61.
Leech bites, 537.
Letter from the senior editor, 176.
Lewis's case of blindness from quinine, 396.
Lightning rods, 364.
Louisville summer school of medicine, 89, 184.
Louisville Medical Institute, 276, 459.
Lungs, carbonic acid exhaled from, 449.
Luxations, 346.

M

Manlove's case of acephalous monster, 401.
Manlove's case of gastrotomy, 250.
Materia Medica, 483.
Medical Society of Tennessee, 90.
Medical Institute of Louisville, 276 459, 548.
Medical Sciences, abstract of, 428.
Medical matters in Geneva, 434.
Medical remembrancer, Shaw's, 125.
Medical university of Alabama, 546.
Medicine, Louisville summer school of, 89, 184, 276.
Medicine, clinical lectures on, 353.
Memphis, convention at, 453.
Mercury, metallic passage of, into the blood, 448.
Mesmeric infirmary, 255.
Mesmerism, true and false, 343.
Metallic mercury, passage of, into the blood, 448.
Meteoric stones, 455.
Meteorological observers, &c., 541.
Midwifery, 518.
Miller's case of phrenitis, 25.
Missouri, typhoid fever in, 88.
Mississippi valley association of dental surgeons, 454.
Modern improvements in surgery, 61.
Morrison on an epidemic in Henderson county, Ky., 198.
Mucous membranes, 471.
Muriate of morphia, poisoning by, applied externally, 436.
Murder—monomania, 368.

N

Naphtha in gout, 449.
Narcotic poisoning, vinegar in, 359.
National medical convention, 233, 308.
Naturalist, 546.
Neil on the arteries, 460.
Neuralgia, treatment of, 326.
Neuroses, 522.
New York medical intelligencer, 547.
New York medical and surgical reporter, 547.
New medical journal, 273, 547.
Nitrate of potash, in spasmodic asthma, 84.

O

Observations on dyspepsia, 164.
Occupation in insanity, 262.
Ohio, diseases of, 546.
Opiates, 511.
Overton on the mucous membranes, 471.

P

Paralysis, 527, 544.
Paralysis of the arm, 344.
Parturition, two cases of, 298.
Pathological anatomy, 542.
Pathology and Therapeutics, 543.
Pericardium, death from abscess bursting into, 267.
Periodicity of neuroses, 522.
Phrenitis, case of, 25.
Physicians of valley of Mississippi,
Poisoning by tartaric acid, 517.
Poisoning by hydrocyanic acid, 258.
Poisoning by cherry kernels, 173.
Poisoning by belladonna, 429.
Poisoning by morphia, applied externally, 436.
Practice of physic, Watson's, 455.
Priapism, case of, 350.
Principles of obstetric medicine, Ramsbotham's, 420,
Prize essay, 90.
Professor Sewall, death of, 89.
Professor Richardson, death of, 361
Professor Cornelian on chlorosis, 174
Professor Dickson on intermarriage of the races, 91.

Prussic acid, poisoning by, 258.
Pulmonary diseases, climate upon, 449.
Q
Quackery, 85.
Quinine, blindness from, 396.
Quinine in diseases of the south, 246.
Quinine, salacine as a substitute for, 275.
R
Ramsbotham's obstetrical medicine, 420.
Ranking's abstract, 428.
Rectum, ulceration of, 268.
Rectum, relaxed, 516.
Richardson's cases of parturition, 298.
Richardson, Prof., death of, 361.
Robards on epidemic erysipelas, 285
Rods, lightning, 364.
Ross's case of injury of the hip, 14.
Rush medical college, 367.
S
Salacine a substitute for quinine, 275.
Scientific discovery, 182.
Secrets of homœopathy, 359.
Senior editor, letter from, 176.
Shaw's medical remembrancer, 425
Simon's chemistry, 497.
Slippery elm bark, bougies of, 433.
Southern and western convention at Memphis, 453.
Spasmodic asthma, nitrate of potash in, 84.
Spontaneous cure of consumption, 270.
Stout's case of gunshot wound, 304
Stackhouse's case of mixed blood, 481.
Stockton's dental intelligencer, 548.
Strychnine in chorea St. Viti, 535.
Strychnia, 544.
Subclavian artery, 536.
Sulphate of quinine in diseases of the south, 246.
Sulphate of quinine, use of, resulting in blindness, 396.
Sulphuric ether, phrenitis from inhalation of, 25.
Surgical cases, Emery's, 410.
Surgery, lectures on, 61, 211.
Surgical operation, case of insanity cured by, 259.
Sutton's case of imperforate vagina, 11.
T
Tartaric acid, 517.
Tennessee, medical society, 90.
Terms used in medicine, by Hoblyn 428.
Tetanus, traumatic, 325.
Therapeutics and materia medica. 459.
Theory and practice of surgery, Cooper's, 420.
Third dentition, case of, 257.
Total blindness from large doses of quinine, 396.
Treatise on diseases of females, by Colombat De l'Isere, 37.
Treatment of mammary abscess by the breast pump, 173.
Treatment of typhus, 264.
Treatment of neuralgia, 326.
Treatment of infantile gastric fever, 328.
Triplets, case of, 258.
Trousseau on anemia, 525.
True and false mesmerism, 343.
Tumor on the thigh, affecting the arterial system, 254.
Typhoid fever in Missouri, 88.
Typhus, treatment of, 264.
U
Ulceration of the rectum, 268.
University, Transylvania, 367.
Urine, absorption of, 345.
Urinary deposits, 329.
V
Vaccination, influence of, 159.
Vagina, case of imperforate, 11.
Veins, admission of air into, &c, 538.
Vestiges of the natural history of creation, 233, 308.
Vinegar in narcotic poisoning, 269.
W
Warm climate in pulmonary diseases, 449.
Watson's lectures on the practice of physic, 455.
West's case of fungous growth of the gums, 405.
Women, diseases of, 530.

### *Receipts of Medical Journal for November, 1845.*

| | |
|---|---|
| Dr. W. R. Snelson, Fayette, Mo., - - - - - - | $10 00 |
| Dr. G. O. Pond, Columbus, Miss., - - - - - | 5 00 |
| Dr. T. B. Albertson, Monroe, Ind., - - - - - | 5 00 |
| Dr. B. F. Russell, New Washington, Ind., - - - - | 5 00 |
| Dr. Z. T. Roberts, Grand Pass, Mo., - - - - - - | 5 00 |
| Dr. Overstreet, St. Josephs, Mo., - - - - - - | 5 00 |
| Dr. W. C. Galt, Louisville, Ky., - - - - - - | 5 00 |
| Dr. J. Drane, New Castle, Ky., - - - - - - - | 5 00 |
| Dr. E. B. Haskins, Clarksville, Tenn. - - - - | 5 00 |
| Dr. A. A. King, Carthage, Tenn., - - - - - - | 10 00 |

### TO SUBSCRIBERS.

We regret to find that, notwithstanding unusual expenditures upon the present volume of this Journal and its very material improvement, our subscribers have this year been more than usually remiss. We would give notice that all who may not remit promptly shall be forthwith and constantly harrassed with every form of dun until they are worried out. Their names shall appear in a black list on the back of the Journal, and, simultaneously, they shall be called upon and persecuted by duns of every kind, travelling agents, local agents, constables, sheriffs, and catchpoles. We are shamefully deprived of our dues and we mean to have justice done us.

☞Receipt by mail, at our risk, postpaid.

PRENTICE & WEISSINGER.

### TRAVELING AGENTS FOR THE WESTERN JOURNAL OF MEDICINE AND SURGERY.

Jno. F. Johnston and Jas. H. Stevenson, Kentucky—C. W. James, Ohio, Indiana, Illinois, Missouri, and Michigan—Geo. C. Brett, Tennessee—Wm. H. Weld & Son, Mississippi and Louisiana.

The Western Journal of Medicine and Surgery is published monthly by the undersigned, at the corner of Main and Fifth streets, Louisville, at $5 per annum, payable in advance. Each number contains 96 pages, making two volumes in the year of more than 550 pages.

Contributions to its pages by the Physicians of the Valley of the Mississippi, addressed to the Editors, are respectfully solicited.

Letters on business, to be addressed, postage paid, to the publishers.

*January*, 1844. PRENTICE & WEISSINGER.

